# MassArray analysis of genomic susceptibility variants in ovarian cancer

**DOI:** 10.1038/s41598-020-76491-7

**Published:** 2020-12-03

**Authors:** Sonali Verma, Indu Sharma, Varun Sharma, Amrita Bhat, Ruchi Shah, Gh. Rasool Bhat, Bhanu Sharma, Divya Bakshi, Ashna Nagpal, Ajay Wakhloo, Audesh Bhat, Rakesh Kumar

**Affiliations:** 1 Indian Council of Medical Research-Centre for Advance Research, Shri Mata Vaishno Devi University, Katra, Jammu and Kashmir India; 2 Ancient DNA Laboratory, Birbal Shani Institute of Paleo Sciences, Lucknow, Uttar Pradesh India; 3 School of Biotechnology, Shri Mata Vaishno Devi University, Katra, India; 4 Department of Biotechnology, Kashmir University, Srinagar, Jammu and Kashmir India; 5 Department of Obstetrics and Gynecology, Government Medical College, Jammu, Jammu and Kashmir India; 6 Centre for Molecular Biology, Central University of Jammu, Jammu, Jammu and Kashmir India

**Keywords:** Cancer genetics, Genotype, Biotechnology, Cancer, Cell biology, Genetics

## Abstract

Ovarian cancer (OC), a multifaceted and genetically heterogeneous malignancy is one of the most common cancers among women. The aim of the study is to unravel the genetic factors associated with OC and the extent of genetic heterogeneity in the populations of Jammu and Kashmir (J&K).Using the high throughput Agena MassARRAY platform, present case control study was designed which comprises 200 histopathological confirmed OC patients and 400 age and ethnicity matched healthy controls to ascertain the association of previously reported eleven single nucleotide polymorphisms (SNPs) spread over ten genes (*DNMT3A, PIK3CA, FGFR2, GSTP1, ERCC5, AKT1, CASC16, CYP19A1, BCL2* and *ERCC1*) within the OC population of Jammu and Kashmir, India. The association of each variant was estimated using logistic regression analyses. Out of the 11 SNPs the odds ratio observed for three SNPs; rs2699887 was (1.72 at 95% CI: 1.19–2.48, *p* = 0.004), rs1695 was (1.87 at 95% CI: 1.28–2.71, *p* = 0.001), and rs2298881 was (0.66 at 95% CI: 0.46–0.96, *p* = 0.03) were found significantly associated with the OC after correction with confounding factors i.e. age & BMI. Furthermore, the estimation of interactive analyses was performed and odds ratio observed was 2.44 (1.72–3.47), p value < 0. 001 suggests that there was a strong existence of interplay between the selected genetic variants in OC, which demonstrate that interactive analysis highlights the role of gene–gene interaction that provides an insight among multiple little effects of various polymorphisms in OC.

## Introduction

Report published by GLOBOCAN in the year 2018 confirm about 18.1 million people were diagnosed with cancer and over 0.7 million are registered in cancer registry every year with estimates deaths of 9.6 million, worldwide^1,2^. In India, the number of new cancer cases in year 2018 were 1.1 million and estimated deaths were approximately 0.75 million^1,3,4^. OC ranks 3rd among females in India after Breast and Cervix cancers^3^. Sixty-one percent of women of J&K (India) are having reproductive health issues like abnormal vaginal discharge, symptoms of a urinary tract infection, pain or bleeding associated with intercourse, uterine endometriosis or even benign and malignant ovarian tumors^5^. It has been observed that there is substantial difference among different Indian populations with special emphasis on their topographical, language, social relationships and genetics^6^.

In the Kashmir valley of North India, OC was third foremost reason of cancer-related deaths in females from 2002 to 2012 (7.45%)^7^. The rise in OC cases in recent past in the population groups of J&K has mandated to identify the reasons including genetic factors that might be responsible for this increased incidence. The population of Jammu and Kashmir is ethnically and genetically diverse compared to the other population groups^8^, thus creating a heterogeneous gene pool. Despite being ethnically diverse, the population groups of J&K have high incidence rate of OC^9^.

There are genetic and environmental factors associated with the risk of OC^10^. Genetics is vitally associated with risk of OC in both sporadic and familial cases^11^. Due to the low mutation rates of *BRCA1* and *BRCA2* genes, they only accounts for fewer cases^12^, leaving a lot of OC cases genetically unidentified. The evaluation of genetic determinants and epidemiologic factors of OC might help in advancing better detection and screening methods^13^. Aggregation of specific genetic alterations, particularly Single Nucleotide Polymorphisms (SNPs) contribute to OC predisposition^14^. SNPs investigated in the present study include genes like *DNMT3A, PIK3CA, FGFR2, GSTP1, ERCC5, AKT1, CASC16, CYP19A1, BCL2,* and*ERCC1.*SNPs of these genes have been found in association with cancers of ovary, breast, stomach and lung among different populations, globally^15–30^. Recently, reported association of *XRCC1* (rs25487), *HoGG1* (rs1052133), *DNAH11*(rs2285947) and *LRFN2*(rs2494938) gene variants with OC provided an insight that genetic variants provide increased risk in the present populations^9,31^. Besides these two reports, no genetic data is available on OC from the J&K region. In order to extend screening, more genetic variants in the J&K population, in-house cancer SNP panel was designed to screen the OC patients that comprises of eleven SNPs of ten genes (*DNMT3A*^17^*, PIK3CA*^21,22^*, FGFR2*^21,22^*, GSTP1*^21,22^*, ERCC5*^18–20^*, AKT1*^16^*, CASC16*^15^*, CYP19A1, BCL2*^15,23,24^*, ERCC1*^25–30^*)*; and population based association study was conducted to assess the genetic predisposition of cancer susceptibility variants with OC.

## Results

The clinical characteristics of both cases and controls are given in Table 1. The mean age of cases was 59.2 (± 10.1) years, which and that of controls was (56.7 ± 14.4) years respectively. The average BMI of the cases (22.6 ± 4.52) was significantly lower than that of the controls (25.4 ± 4.89) (*p* = 9.74E − 12).

**Table 1:** Clinical details of cases and controls.

Characteristics	Cases (200)	Controls (400)	p value
Age (years) Mean ± SD	59.2 ± 10.1	56.7 ± 14.4	0.02
BMI Mean ± SD	22.6 ± 4.52	25.4 ± 4.89	9.74E−12
**Menopausal status**			
Premenopausal	124	276	0.33
Post-menopausal	74	124	
**Stage**			
I/II	78	–	–
III/IV	110	–	
**Age at menarche (years)**			
> 12	107	215	0.02
< 12	93	185	
**Histology of tumors**			
Epithelial	123	–	–
Endometroid	15		
Germ cell	9	–	
Sex cord stromal cell	33	–	
Metastasis	20	–	
**Oral contraceptive use**			
Yes	80	–	–
No	120	–	
**Breast nodules**			
Yes	22	–	
No	162	–	

Out of 11 SNPs genotyped, only six SNPs (rs2699887, rs1695, rs2298881, rs10046, rs2981582, and rs751402) were having genotyping quality of more than 95% following stringent quality check and hence were included in the analyses. The allelic frequency distribution of these six SNPs in cases and controls are given in Table 2. Out of these six SNPs, only rs2699887, rs1695, and rs2298881 were found significantly associated with OC in the studied population. The observed allelic Odds Ratio (OR) of rs2699887was (1.5 at 95% CI: 1.1–2.0, *p* = 0.003), rs1695 was (1.4 at 95% CI: 1.0–1.8, *p* = 0.01) and rs2298881 was (0.6 at 95% CI: 0.4–0.9, *p* = 0.03), respectively. The observed allelic OR for the non-significantly associated SNPs rs10046of *CYP19A1*, rs2981582 of *FGFR2*, and rs751402of *ERCC5* was (1.2 at 95% CI: 0.9–1.5, *p* = 0.12), (1.1 at 95% CI: 0.8–1.4, *p* = 0.33), and (0.8 at 95% CI (0.6–1.1), *p* = 0.21), respectively.

**Table 2:** Distribution of risk allele frequency and association analyses of variants with genotyping call greater than 95%.

S.No	Gene	SNPs	Frequency in cases	Frequency in control	HWE	OR 95% CI	p value	OR* 95% CI (dominant model)	p value * (dominant model)	PAR
1	CYP19A1	rs10046	A = 0.3447	A = 0.3	0.6271	1.2 (0.9–1.5)	0.12	1.36 (0.946–1.97)	0.95	–
			G = 0.6553	G = 0.7						
2	PIK3CA	rs2699887	T = 0.259	T = 0.185	0.0682	1.5 (1.1–2.0)	0.003	1.72 (1.19–2.48)	0.004	25.93
			C = 0.741	C = 0.815						
3	FGFR2	rs2981582	A = 0.3613	A = 0.3325	0.4957	1.1 (0.8–1.4)	0.33	1.30 (0.89–1.88)	0.165	–
			G = 0.6387	G = 0.6675						
4	GSTP1	rs1695	G = 0.2539	G = 0.1941	0.872	1.4 (1.0–1.8)	0.01	1.87 (1.28–2.71)	0.001	39.2
			A = 0.7461	A = 0.8059						
5	ERCC1	rs2298881	A = 0.2356	A = 0.3029	0.9036	0.7 (0.53–0.94)	0.01	0.66 (0.46–0.96)	0.03	–
			C = 0.7644	C = 6971						
6	ERCC5	rs751402	A = 0.2216	A = 0.2552	0.5037	0.8 (0.6–1.1)	0.21	0.71 (0.48–1.03)	0.07	–
			G = 0.7784	G = 0.7448						

*Adjusted with age and BMI.

After applying logistic regression in order to avoid any biasness caused by confounding factors like age and BMI, all three significantly associated variants followed the same direction of association. Adjusted OR of rs2699887 was (1.72 at 95% CI: 1.19–2.48, *p* = 0.004), rs1695 was (1.87 at 95% CI: 1.28–2.71, *p* = 0.001), and rs2298881 was (0.66 at 95% CI: 0.46–0.96, *p* = 0.03). The SNPs that were not included in the final analyses because of their low call rate (< 95%) are summarized in Supplementary data: table S3.

Interaction analysis between the three significantly associated SNPs revealed that two SNPs were risk alleles with 10/10 cross-validation consistency (CVC) and one SNP had a Testing Balance Accuracy (TBA) score of above 0.50 in all the SNPs Table 3.

**Table 3:** Interaction analysis OC cases and controls.

SNP combination	Cross-validation statistics		p value
SNP1_rs2699887, SNP2_rs1695, SNP3_rs2298881	Balanced accuracy	0.605	< 0.0001
	Accuracy	0.64	
	Specificity	0.71	
	Odds ratio	2.4483 (1.7229–3.4791)	

*p < 0.05 was considered significant.

**Figure 1:**
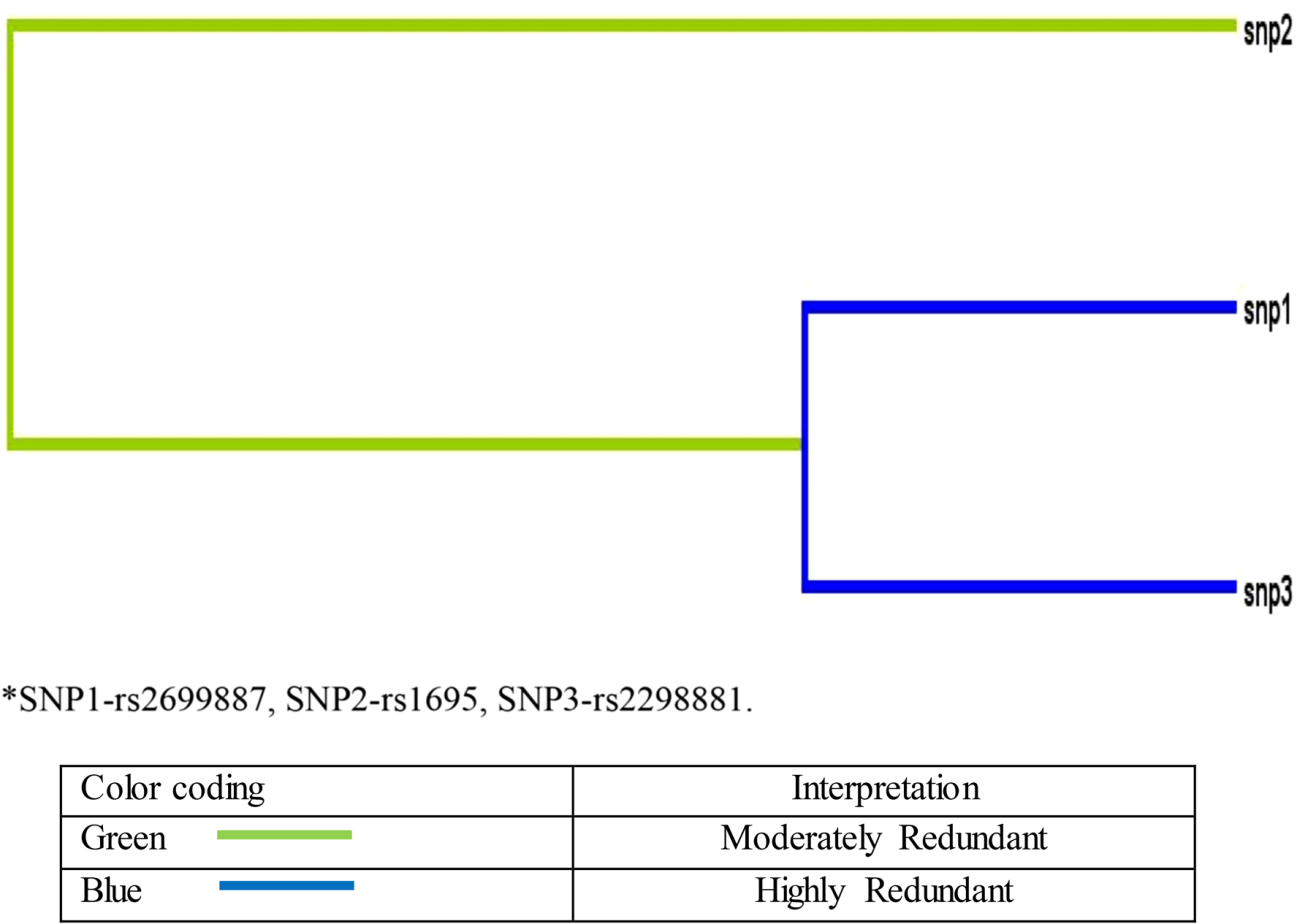
SNP-SNP interaction analysis using MDR, color-coding of bars used to interpret interactions.

Entropy dendrogram revealed that SNP rs1695has a synergistic effect on SNPrs2699887 and SNP rs2298881. However, strong redundancy was observed between SNP rs2699887 and SNP 3rs2298881 Fig. 1. Interactive analyses revealed that there are interactions between genetic variants of *PIK3CA*, *GSTP1* and *ERCC* genes [OR-2.4483 (1.7229–3.4791), *p* > 0.0001] via permutation test of 1000 iterations Table 3. Sample size included in the present study had 80–90%, power assuming minor allele frequency 0.20 to detect the association with O.R (1.2–1.7).

### Functional annotation of associated SNPs

As per the UCSC Genome browser database (http://genome.ucsc.edu/), SNPs rs2699887, rs1695 and rs2298881 fall in the regulatory region which was generated from Epigenome, Encyclopedia of DNA Elements (ENCODE) project where all the associated SNPs (rs2699887, rs1695 and rs2298881) are located nearby the enhancer region (H3K4Me1 mark) and are also observed near the active promoter region (H3K27Ac mark) as well as in seven cell lines (H1-hESC, GM12878, K562, HUVEC, HSMM, NHLF, and NHEK) which specified that SNPs (rs2699887, rs1695 and rs2298881) were possibly intricate with the gene expression, whereas SNPs (rs2699887, rs1695 and rs2298881) also knockdown into 125 types of cells in DNase I hypersensitive region (Supplementary data: figure S2, S3 & S4). The transcription factor data from ENCODE and haploreg of associated SNPs rs2699887, rs1695 and rs2298881 also indicates that these variants alter certain regulatory motifs (Supplementary data: figure S1).The role of rs2699887, rs1695 and rs2298881 SNPs; were also examined by using current biological servers (GTEX^32^, Haploreg^33^). We assessed the gene expression of tissues using data from GTEX portal and found that the eQTL expression of ovarian tissue of variant rs1695 of *GSTP1* significant association with change in gene *NDUFV1,* showing change in expression of ovarian tissue with *p* value = 0.0000018 and NES value is 0.45 and variant rs2298881 of *ERCC1* confirm expression change of ovarian tissue with p value = 7.9E − 11 and NES value = − 0.48 (reduced effect).

Therefore, we could speculate that functional SNPs located in PI*K3CA* and *GSTP1* gene may disrupt transcription factor response elements, and further affect the expression level of *PIK3CA* and *GSTP1* and ultimately affect the occurrence and development of ovarian cancer. On the basis of UCSC genome browser, haploreg and GTEX eQTL data for ovarian cancer. The study also found that rs2699887 and rs1695 were associated with the prognosis of ovarian cancer after adjustment for age and BMI.

These findings proved that functional annotation of risk associated variants rs2699887 and rs1695 were differentially regulated in ovarian tissues which suggested a possible mechanism for the effect on ovarian cancer risk. However, risk estimates suggested that *ERCC1* rs2298881is a protection-associated genetic variation in ovarian cancer, whereas no association was found for rs10046, rs2981582 and rs751402.

## Discussion

PI3K/AKT pathway under which the *PIK3CA* gene belongs; plays an important role in cell cycle progression, programmed cell death and drug resistance. Consequently, various SNPs in the *PI3K/AKT* pathway genes were found associated with different malignancies. The rs2699887 (C > T) variant in the upstream region of intron-1 has been associated with various cancers including OC^34–36^. This variant has also been associated with lung cancer toxicity in patients who underwent platinum-based chemotherapy^37^. Allele ‘T’ of variant rs2699887 was found associated with increased susceptibility towards OC. Interestingly, this variant has been reported to cause transcription factor binding effect which leads to change in normal splicing patterns^34^. The role of this variant in OC and other cancer susceptibility may be due to diverse role of *PIK3CA* in the initiation and progression of OC. The mutation in *PIK3CA* gene have been frequently identified in endometrial ovarian carcinomas and not found in serous epithelial ovarian carcinomas^38^. Over-expression of *AKT* gene from *PI3K/AKT* pathway may leads to progression and metastasis of OC^34,39^. The association of rs1695 (A > G) variant with OC is the first studied population in India. It was previously associated with OC in Brazilian, Australian and French populations but not in Caucasian and Chinese populations^22,40,41^. The rs1695 (A > G) variant, missense mutation in exon 5 of *GSTP1* that changes amino acid 105 from isoleucine to valine(I105V). The I105V change has prognostic effect in OC patients with paclitaxel plus carboplatin combination chemotherapy (TC therapy)^21^. Additionally, in some populations the mutant and wild alleles defend cells against programmed cell death through JNK pathway and wild type allele provided risk in epithelial OC risk^42^. In addition to the significant association of ‘AA’ genotype of rs1695 with OC, we also identified that rs2699887 and rs1695 variants in combination increases risk of OC in post-menopausal women than pre-menopausal women. Thus, indicating that *GSTP1* and *PIK3CA* mutants when in combination are highly responsible for development of OC in studied population group.

Published data regarding rs2298881 in the *ERCC1*gene and rs751402 in the *ERCC5* gene with OC although scanty, retrospective studies have associated rs2298881 and rs751402 with increased risk for breast, lung, colorectal, gastric and several other types of cancers^25,27,43,44^. Contrary to this, our data supports a protective nature of rs2298881 variant in the studied population. Variant rs751402 of *ERCC5* on the other hand was not found associated with OC. These results were consistent with our previous report^45^, whereas another polymorphisms of DNA repair pathway rs25487 (*XRCC1*) was significantly not associated with OC.

We excavated the following database (Epigenome, ENCODE-UCSC genome browser, haploreg, GTEX mobile portal) and observed that rs2699887, and rs1695 and rs2298881 could affect the gene expression and are likely to modify regulatory motifs and disrupt protein binding activities (Supplementary data: figure S1, S2, S3 and S4). All three SNPs (rs2699887, rs1695 and rs2298881) in ENCODE data fall in the hypersensitivity region of DNAse, in enhancer (H3K4Me1) and in promoter (H3K27Ac) region in seven cell lines from Epigenome and ENCODE, suggesting that there was a probable mechanism which effected the regulation of gene expression resulting in OC risk.

In the light of significant association of three variants either directly or indirectly with OC in the studied population; we further evaluated these variants in GTEX mobile portal. The rs1695 variant showed eQTL expression with *NDUFV1* gene in ovarian tissue indicating the change in expression in *NDUFV1* gene, where *NDUFV1* gene plays a role in apoptosis due to reactive oxygen species (ROS) which may have strong implication on OC^46^. The rs2298881 variant showed eQTL expression in ovarian tissues indicating the change in expression.

In summary, our study revealed that SNP rs2699887 of *PIK3CA,* rs1695 of *GSTP1* and rs2298881 of*ERCC1* gene might affect the expression directly or indirectly in OC and ultimately modify the OC risk in J&K. The findings support for additional functional studies to identify the biological mechanism behind the progression of OC. To the best of our knowledge this is the prelude study that has replicated the association between various polymorphisms and the risk of OC in J&K women; however, the functional validation of these variants is mandatory in order to better understand the association between these variables and its possible role in OC.

## Conclusion

The observation from the study highlights the role of independent as well as interactive effect of SNPs in OC. The interactive analysis also provides insight that variants of *PIK3CA*, *GSTP1* and *ERCC1* have relatively high risk of OC. Hence, more replication studies with larger sample size are required along with their functional validations to unravel the biological significance of SNPs in OC.

## Materials and methodology

### Ethics statement

This case–control genetic association study was approved by the Institutional Ethical Review Board (IERB) of Shri Mata Vaishno Devi University under notification number (SMVDU/IERB/16/48). All experimental protocols were conducted according to the guidelines and regulations set by the Institutional Ethical Review Board (IERB).

### Sampling of subjects

A total of 600 participants (200 histopathological confirmed OC patients and 400 healthy females) were included in the study with written informed consent. The participant details were duly filled pro forma and was signed by the participants. 2–3 ml of venous blood was collected in EDTA vials. Samples were collected from various hospitals and clinics of J&K region. The clinical parameters are listed in Table 1.

### SNP selection

In the study, the variants were selected on the basis of following criteria:

1. The potential genetic variation data implicated in carcinogenesis and associated traits were retrieved from the NCBI’s Single Nucleotide Polymorphism database (dbSNP-NCBI), including the variants previously associated with OC and other cancers.
2. Only variations having annotation inexonic promoter region, 5′ un-translated regions (5′ UTR) or 3′ UTR, exonic and intronic SNPs (if the condition or criteria for SNP selection were met) were screened. The details of selected SNPs are given (Supplementary data: Table S1.

### Genotyping

The genomic DNA was isolated by using the Qiagen DNA isolation kit (Catalogue No. 51206). Genotyping was performed using Agena MassArray platform, a robust and highly sensitive tool for genotyping of SNPs^47^ available in the Central Analyzer Mass Array facility at SMVDU. The SNP panel was customized by using Agena Bioscience Assay Design Suite (version 2.0). The sequence of primers have been provided in (Supplementary data: Table S2).The whole methodology for pool PCR, Shrimp Alkaline Phosphatase (SAP) and iPLEX PCR was adopted from studies^47,48^. The genotyping results were validated by replicating 10% of random samples and the concordance rate was 98.3%. In the reaction of 384 well plates, one negative and one positive control were added in every reaction to check the quality of the reaction.

### Genotyping quality control

For genotyping quality assurance, the following criteria were applied:(i) SNPs having call rate > 95% were included in the statistical analysis^49^ and(ii) Hardy–Weinberg Equilibrium (HWE) among cases and controls was used for assessing the quality of genotypes.

### Statistical analysis

Statistical analyses was mainly performed using Plink V.1.09^50^ with maximum 10,000 permutations. Significance of the association was evaluated by 3 × 2 chi square test. Logistic regression analysis was performed using SPSS V.23 in order to obtain corrected odds ratio (OR), confidence interval (CI) and p-value from confounding factors like age and BMI. The SNP-SNP interaction to analyze the synergic effect of significantly associated SNPs was performed by using Multifactor Dimensionality Reduction (MDR) software^51^. Population attribution risk (PAR) percentage was also estimated for significantly associated risk variants by using adjusted OR. Power of the study was estimated by using CATS online calculator^52^.

### Putative analysis of SNPs

The study also interpreted the candidate SNPs in regulatory region assembled in Epigenome (https://www.roadmapepigenomics.org)^53^, Encyclopedia of DNA Elements (ENCODE) tool from UCSC Genome Browser (https://www.UCSCgenomebrowser)^54^. The present study also investigated the data of several cell lines (H3K4Me3, H3K27Ac, and H3K4Me1) with special emphasis on H1-hESC, GM12878, K562, HUVEC, HSMM, NHLF, and NHEK cells. Further, the study examined DNaseI Hypersensitivity region and transcription factor binding sites including their changed motifs from Haploreg (https://www.haploreg.org)^33^ data 55in 125 cell types. Genotype-Tissue Expression (GTEx) portal was used for the identification of gene expression of our candidate SNPs of various genes in ovarian tissue (https://www.gtexportal.org)^55^.

## Supplementary information

Supplementary Information.
