# Supplementary material for: MassArray analysis of genomic susceptibility variants in ovarian cancer
Source: Sci Rep. 2020 Dec 3;10:21101. doi: 10.1038/s41598-020-76491-7 (PMC7713113; doi:10.1038/s41598-020-76491-7)
Supplement: Supplementary file 1 — Supplementary Information. [file 41598_2020_76491_MOESM1_ESM.docx]

**MassArray Analysis of Genomic Susceptibility Variants in Ovarian Cancer**

**Sonali Verma^1,3*^,** Indu Sharma^2^, Varun Sharma^2^, Amrita Bhat^3^, Ruchi Shah^4^, Gh. Rassol Bhat^3^, Bhanu Sharma^3^, Divya Bakshi^3^, Ashna Nagpal^3^**,** Ajay Wakhloo^5^, Audesh Bhat^6^, and Rakesh Kumar^1,3*^

1. Indian Council of Medical Research-Centre for Advance Research, Shri Mata Vaishno Devi University -Katra J&K, India, Email: Sonali.verma@smvdu.ac.in
2. Ancient DNA Laboratory, Birbal ShaniInstitute of Paleosciences Lucknow, UP, India. Email: induadmo@gmail.com and sharmavarun840@gmail.com
3. School of Biotechnology, Shri Mata Vaishno Devi University, Katra, India

Email: amritabhatt88@gmail.com and seithbhat11@gmail.com, bhanugaur91@gmail.com,divya.bakshi09@gmail.com and ashna.nagpal.phd@gmail.com

1. Department of Biotechnology, Kashmir University, J&K, India,

Email: scientistdobt@gmail.com

1. Department of Obstetrics and Gynecology, Government Medical College Jammu, J&K, India. Email:drajaywakhloo@gmail.com
2. Centre for Molecular Biology, Central University of Jammu, J&K, India.

Email: audeshs2002@gmail.com

***Corresponding authors**

Dr. Sonali Verma, Ph.D.

Scientist,ICMR-CAR, School of Biotechnology

Shri Mata Vaishno Devi University, Katra, Jammu and Kashmir, India

Email: Sonali.verma@smvdu.ac.in

&

**Dr. Rakesh Kumar, Ph.D.**

ICMR-CAR, School of Biotechnology,

Shri Mata Vaishno Devi University, Katra, Jammu and Kashmir, India

Email: drrakeshthusoo@gmail.com

**Supplementary Figures**


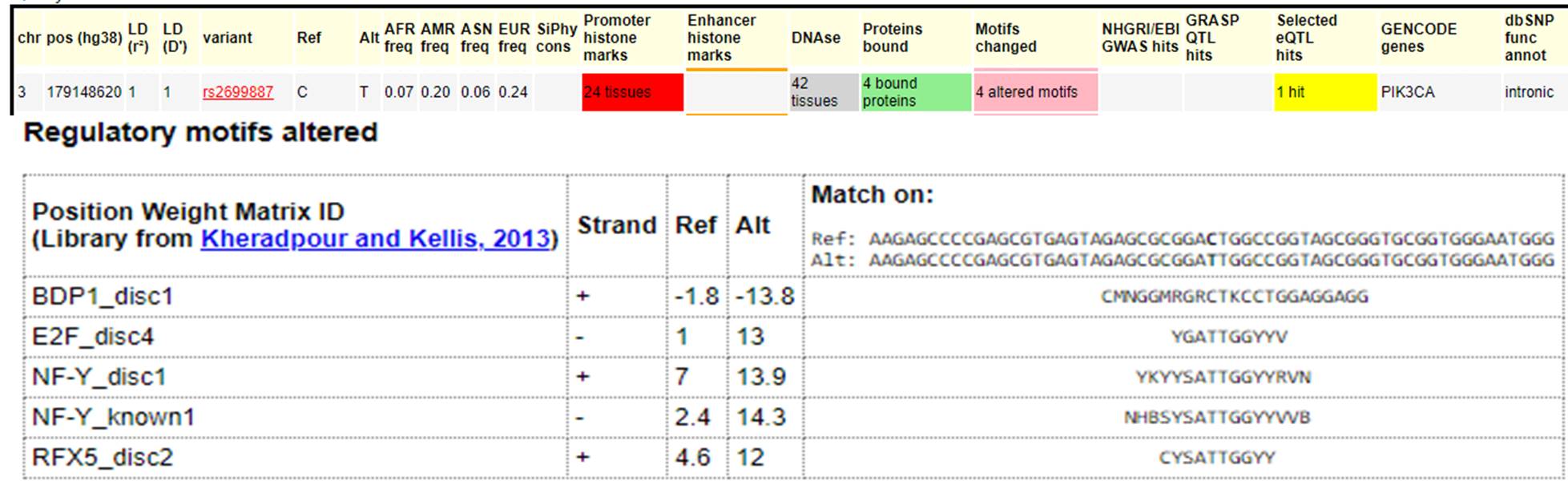


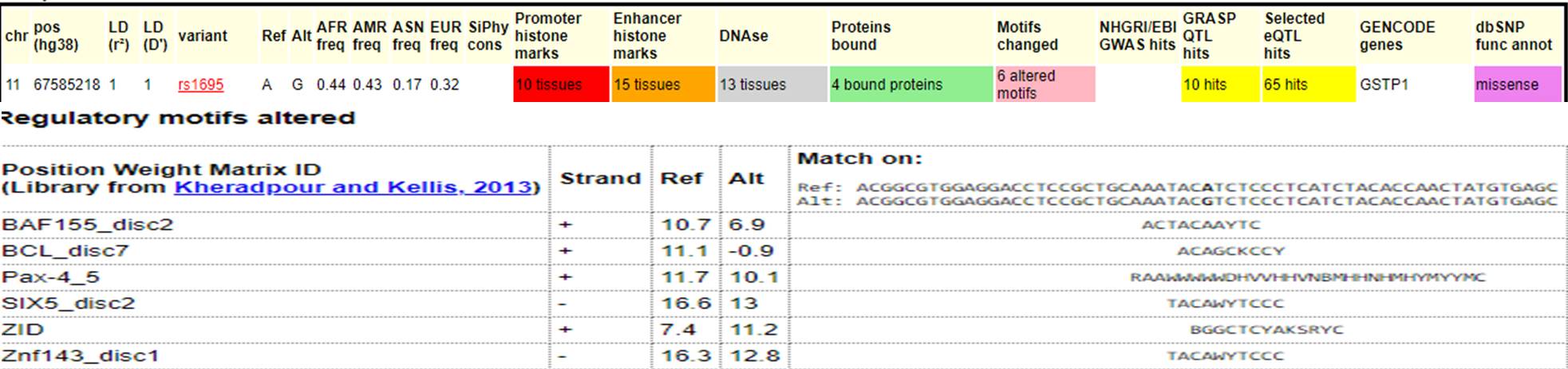


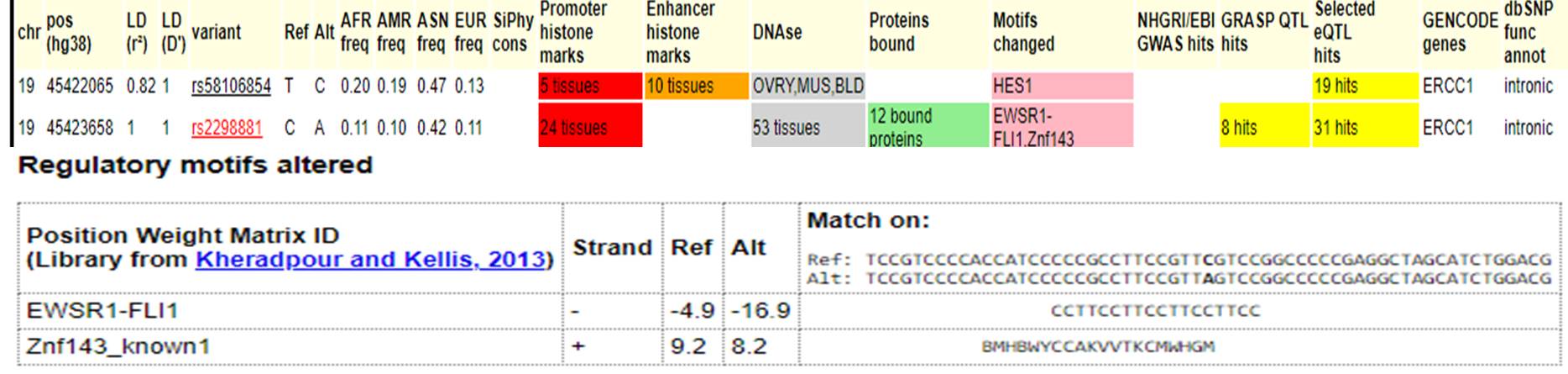


**Supplementary Figure S1.** Representation of motifs changed for the variants rs2699887 *PIK3CA,* rs1695 *GSTP1* and rs2298881 *ERCC1* could affect their corresponding gene expression from Haploreg (www.haploreg.com)^1^.


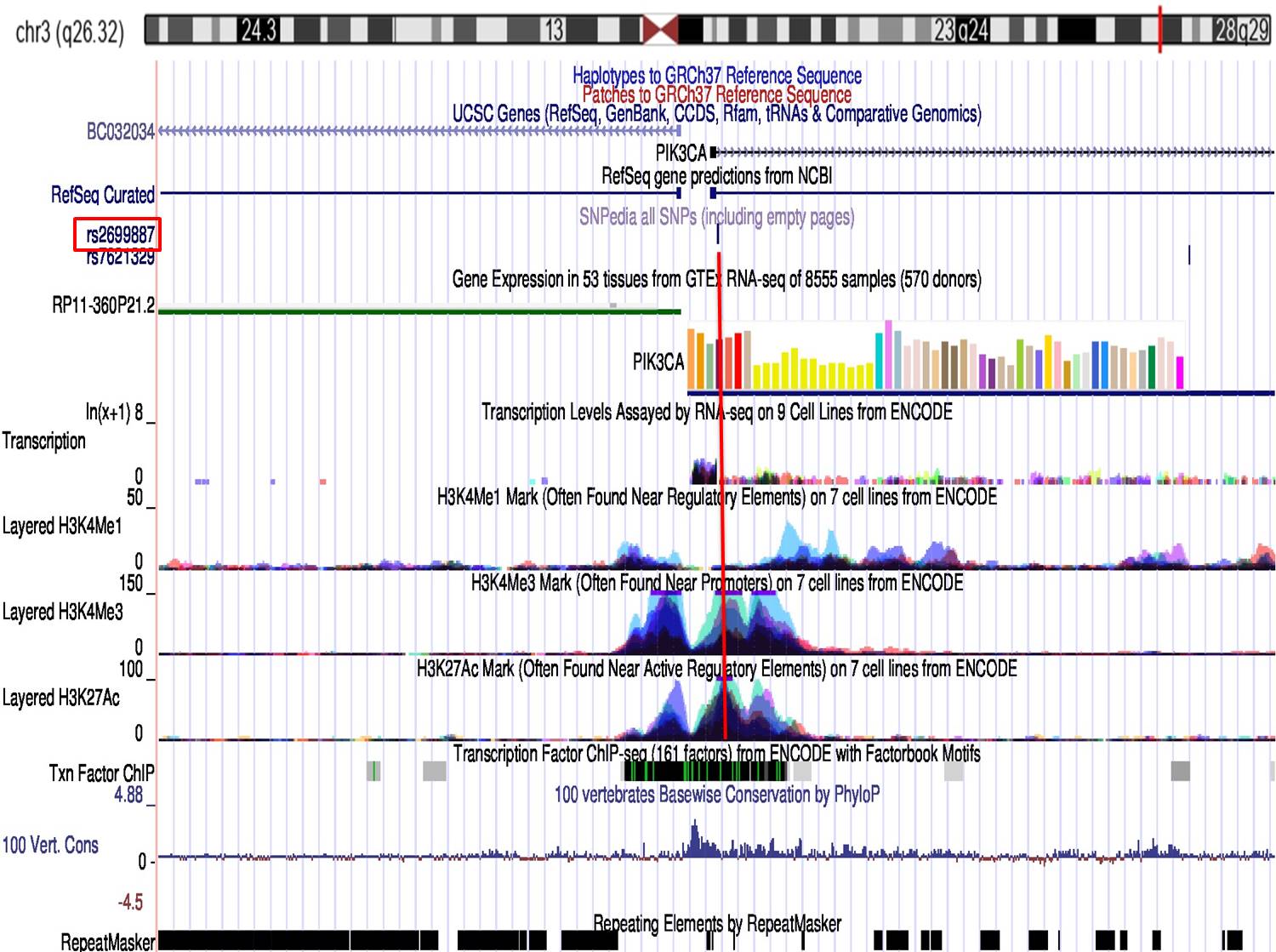


**Supplementary Figure S2. Functional annotation of SNP rs2699887 in *PIK3CA* location by using Encyclopedia of DNA Elements (ENCODE) tool from UCSC Genome Browser (www.UCSCgenomebrowser)^2^.** CHIP-seq tracks for promoter histone marks (H3K4Me3) and enhancer histone marks (H3K4Me1, H3K27Ac) in seven cell lines (GM12878, H1-hESC, HSMM, HUVEC, K562, NHEK, and NHLF cells) are present along with DNase hypersensitivity tracks from ENCODE. The red color line indicates the intronic upstream (2KB) position of SNP **rs2699887**.


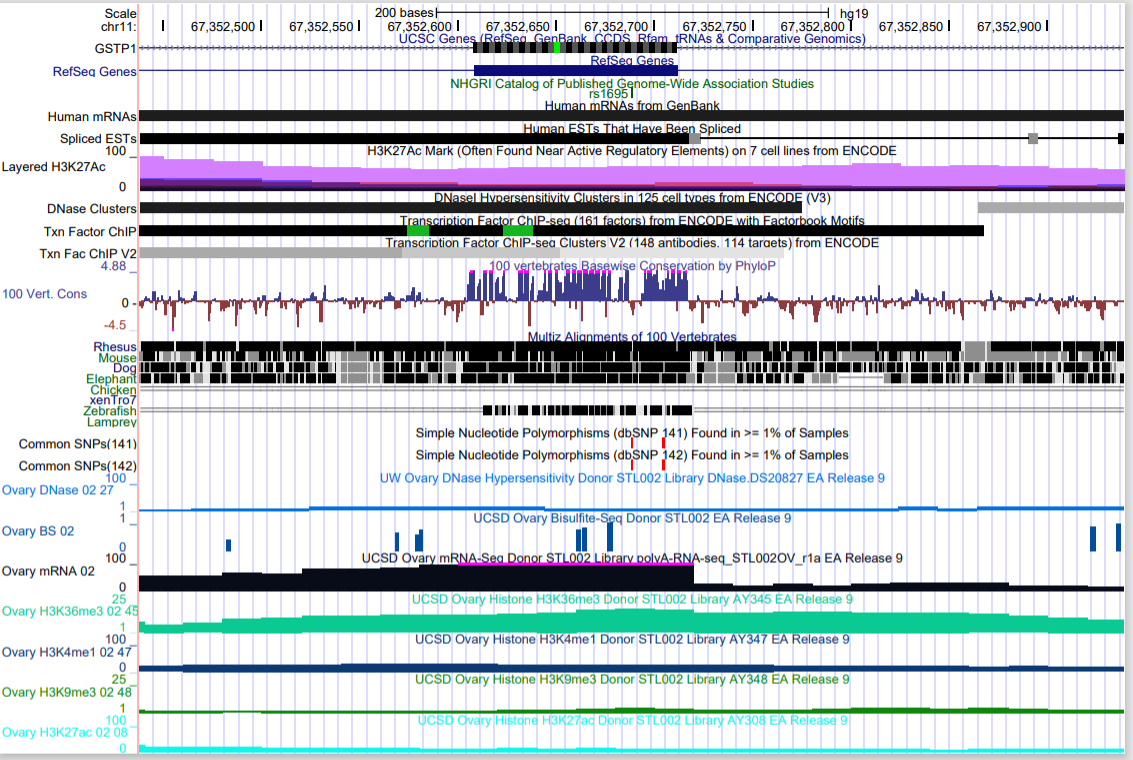


**Supplementary Figure S3. Functional annotation of SNP rs1695 in *GSTP1* location from Epigenome (www.roadmapepigenomics.org)^3^.** CHIP-seq tracks for promoter histone marks (H3K4Me3) and enhancer histone marks (H3K4Me1, H3K27Ac) in seven cell lines (GM12878, H1-hESC, HSMM, HUVEC, K562, NHEK, and NHLF cells) are present along with DNase hypersensitivity tracks from Encyclopedia of DNA Elements (ENCODE).


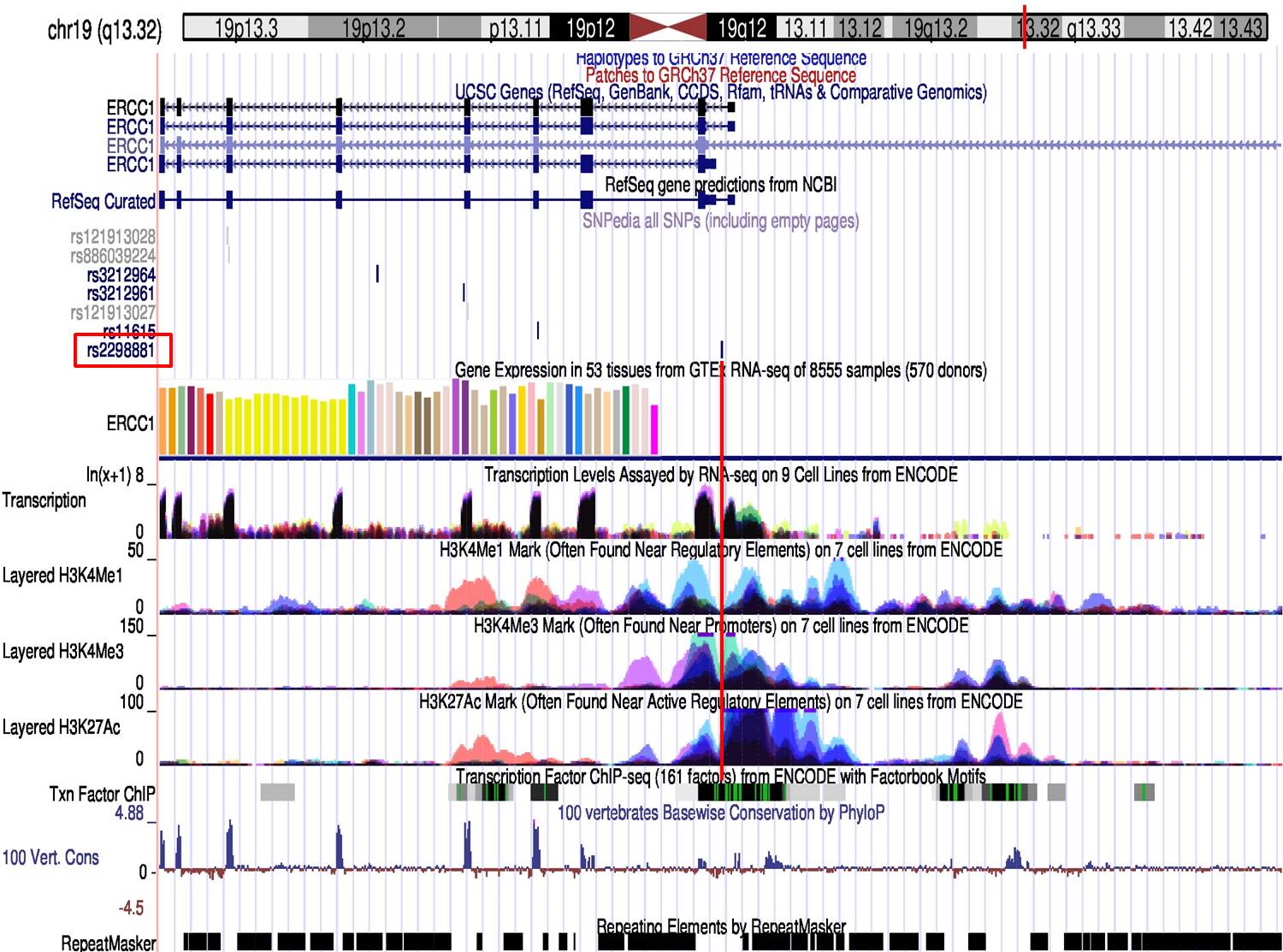


**Supplementary Figure S4. Functional annotation of SNP rs2298881 in *ERCC1* location by using Encyclopedia of DNA Elements (ENCODE) tool from UCSC Genome Browser (www.UCSCgenomebrowser)2.** CHIP-seq tracks for promoter histone marks (H3K4Me3) and enhancer histone marks (H3K4Me1, H3K27Ac) in seven cell lines (GM12878, H1-hESC, HSMM, HUVEC, K562, NHEK, and NHLF cells) are present along with DNase hypersensitivity tracks from Encyclopedia of DNA Elements (ENCODE). The red color line indicates the intronic upstream (2KB) position of SNP **rs2298881**.

| **Supplementary Table S1: Characteristics of 11 variants from 10 genes** | | | | | |
| --- | --- | --- | --- | --- | --- |
| **SNP** | **GENE** | **CHANGE** | **POSITION** | **FUNCTION ANNOTATION** | **ROLE OF GENE AND THEIR SELECTED SNP** |
| rs10046 | CYP19A1 | C>T | 15:51210789 | intron variant variant 3 prime | CYP19A1 encrypts an adherent of the cytochrome P450 super family of enzymes (aromatase). The enzyme aromatase encoded by CYP19A1 helps in synthesis of estrogens from androgens. The process of conversion of androgens to estrogen mostly occurs in the ovaries of premenopausal women. Previous studies found a candidate SNP variant of CYP19A1 (rs10046) has been found to be associated with breast, lung and other cancers^4-6^. |
| rs2699887 | PIK3CA | C>T | 3:179148620 | intron variant, upstream variant 2KB | PI3K/AKT pathway is activated by growth factors ^7^. It is proved that the level of estrogens and PI3K interacts through non-transcription pathway^8^. The phosphatidylinositol 3 kinase (PI3K) pathway is frequently altered in OC by activating the mutation of catalytic subunit of p110α of PIK3CA^9^. The amplification and mutation of PIK3CA gene have been frequently identified in endometrial ovarian carcinomas and not found in serous epithelial ovarian carcinomas^10^. |
| rs2981582 | FGFR2 | A>G | 10:121592803 | intron variant | In FGFR signaling the expression of FGF is tissue specific where the disturbed paracrine signaling of FGFR may help in cancer cell proliferation^11^. It was proved that the expression of FGFR2 effects survival of OC patients who have under gone platinum based chemotherapy by enhancing the chemo sensitivity^12^ Whereas, same results were found in Cole et al^13^study, they proves the strong association between the expression of FGFR2 and platinum based drugs effects (naïve, sensitive and resistant) in OC patients with inhibited effect of cell proliferation. |
| rs1695 | GSTP1 | A>G | 11:67585218 | missense | Glutathione S-transferase P1 have been found to play a role in cell progression where Glutathione S-transferase P1 reduced activity also leads to enhanced JNK activity in OC cells^14^. It was found that many other genes of RAS/ MAPK pathway were up & down regulated due to deletion of GSTP1 gene^15^. In patients who have under gone platinum based chemotherapy, Glutathione S-transferase P1 can decrease the effect of carboplatin which interacts with DNA because the Glutathione S-transferase P1 is generally involved in cellular defense. The polymorphic missense variant rs1695 of GSTP1 has been found to be associated with ovarian as well as breast cancer^16,17^. |
| rs2298881 | ERCC1 | C>A / C>T | 19:45423658 | intron variant, upstream variant 2KB | ERCC Excision Repair 1 (ERCC1) is an Endo-nuclease non-Catalytic subunit of non- homologous excision repair (NER). Mutation in NER pathway affects the genome stability which enhances the chance of developing cancer^18^. In humans the NER is composed of various steps (to identify DNA damage, slice the lesion and repair and attach the patch), except this in OC patients the platinum-based drugs (cisplatin& carboplatin) used in chemotherapy treatments where platinum makes cross links between purines present in double strand DNA by leaving adducts. This adducts formation is removed by Non- homologous excision repair mechanism with their genes (ERCC) by making complex with XPF (ERCC/XPF) which leads to removal of DNA adducts and repairs the damaged DNA, thus escaping apoptosis^19^.This makes ERCC1 sensitivity against platinum based drugs in OC patients, |
| rs11615 |  | A>G | 19:45420395 | nc transcript variant, synonymous codon |  |
| rs751402 | ERCC5 | A>G | 13:102845848 | utr variant 5 prime | ERCC Excision Repair 1 (ERCC5) is an Endo-nuclease non-Catalytic subunit of non- homologous excision repair (NER). It also act as a novel biomarker in OC patients; it makes patients more sensitive to cisplatin with progression free survival^20^. [Christine S. Walsh](http://ascopubs.org/author/Walsh%2C+Christine+S)et al ^21^observed thatin OC patients the platinum-based drug (cisplatin& carboplatin) increases the sensitivity only in the presence of down regulated expression of ERCC5. Thus, the uses of platinum-based drugs in treatment of cancers are frequently restricted due to increase in resistance. The intronic variant rs2298881 and UTR’5 variant rs751402 has been found to be associated with various cancers^18,22-25^ |
| rs2289195 | DNMT3A | G>A | 2:25240614 | intron variant | Dnmt1 and Dnmt3a are important DNA methyltransferases |
| rs2494752 | AKT1 | A>G | 14:104797271 | upstream variant 2KB | The SNP rs2494752 were found to be associated with increased cancer risk, SNP rs2494752 is found to be located at UTR’5 region of AKT1 gene which regulates the transcription and translation of AKT1 gene lead to progression of cancer^26^. Estrogen induced up-regulated expression of AKT and down-regulated expression of nm23-H1 leads to cell invasion in ovarian carcinoma. So, it is suggested that the AKT expression in serous ovarian carcinoma becomes a negative prognostic factor whereas expression of nm23-H1 helps in prognosis in ovarian carcinoma patients^27^. |
| rs3803662 | CASC16 | A>G | 16:52552429 | nc transcript variant | The hypothetical gene (LOC643714) located at chromosome 16 which was recently named as cancer-susceptibility candidate 16 (CASC16). ^28^ |
| rs1564483 | BCL2 | C>T | 18:63127421 | utr variant 3 prime | BCL2 play their role in apoptosis dys-regulation and proliferation of malignant cells.^29,30^. |

**Supplementary Table S2: List of sequence of Primers and UEPs of the Selected Variants for Mass ARRAY Genotyping.**

| **S. No** | **Gene** | **SNP** | **Forward Sequence** | **Reverse Sequence** | **Universal Extended primer (UEP)** |
| --- | --- | --- | --- | --- | --- |
| 1 | rs10046 | CYP19A1 | ACGTTGGATGGACACTATTGGCAAGGATGG | ACGTTGGATGTGGAACACTAGAGAAGGCTG | cccgGAGAAATGCTCCAGAGT |
| 2 | rs2699887 | PIK3CA | ACGTTGGATGTGGGACCCGATGCGGTTAGA | ACGTTGGATGATTCCCACCGCACCCGCTA | gGTGAGTAGAGCGCGGA |
| 3 | rs2981582 | FGFR2 | ACGTTGGATGACTGCTGCGGGTTCCTAAAG | ACGTTGGATGGCACTCATCGCCACTTAATG | CCACTTAATGAACCTGTTTG |
| 4 | rs1695 | GSTP1 | ACGTTGGATGTGGTGGACATGGTGAATGAC | ACGTTGGATGGCAGATGCTCACATAGTTGG | acacAGTTGGTGTAGATGAGGGAGA |
| 5 | rs2298881 | ERCC1 | ACGTTGGATGATTCTATTGGCTCCGTCCCC | ACGTTGGATGAGAGATGGACAAGGCCAGG | gtcaCCACCATCCCCCGCCTTCCGTT |
| 6 | rs11615 | ERCC1 | ACGTTGGATGATAGTCGGGAATTACGTCGC | ACGTTGGATGTTGATGGCTTCTGCCCTTCG | TGAAGTTCGTGCGCAA |
| 7 | rs751402 | ERCC5 | ACGTTGGATGGTATTAGACGGAAACCGAGC | ACGTTGGATGAAACAGCCAGAAGATGTCCC | cGCGGGCCCATTTTTC |
| 8 | rs2289195 | DNMT3A | ACGTTGGATGGGGCAGAAATATCCAAGGAG | ACGTTGGATGACGGTAGGTACCATCCTGTC | gaGAAGCACCAGCTGAGAA |
| 9 | rs2494752 | AKT1 | ACGTTGGATGGGATGGAGGAAGGAATTCAG | ACGTTGGATGTTGCTGGCCTTGTCTCATGG | GAGGAAGCAGAGGGT |
| 10 | rs3803662 | CASC16 | ACGTTGGATGTTTCTTCGCAAATGGGTGGG | ACGTTGGATGTTTTCTCTCCTTAATGCCTC | ATGCCTCTATAGCTGTC |
| 11 | rs1564483 | BCL2 | ACGTTGGATGCCTTCCCTCTACAGTGATAC | ACGTTGGATGCGTTTTCACGTGGAGCATGG | ctcGATACATGTCTTAAGAAGGGT |

**Supplementary Table S3: Allele frequency, odds ratio of SNPs (call rate <95)**

|  |  | | | | | | | |
| --- | --- | --- | --- | --- | --- | --- | --- | --- |
| **S.No.** | **SNP** | **GENE NAME** | **Call rate** | **Cases** | **Control** | **H.W.E, p VALUE** | **ODDS RATIO AT 95% CI** | **P VALUE** |
| **1** | rs2289195 | DNMT3A | 87.8 | A=0.3645 | A=0.3292 | 0.1888 | 1.16(0.89-1.53) | 0.26 |
|  |  |  |  | G=0.6355 | G=0.6708 |  |  |  |
| **2** | rs2494752 | AKT1 | 87.8 | A=0.2471 | A=0.2626 | 0.5839 | 0.92(0.68-1.24) | 0.58 |
|  |  |  |  | G=0.7529 | G=0.7374 |  |  |  |
| **3** | rs3803662 | CASC16 | 84.5 | A=0.906 | A=0.25 | 0.11 | 0.70(0.50-0.98) | **0.03** |
|  |  |  |  | G=0.8094 | G=0.75 |  |  |  |
| **4** | rs1564483 | BCL2 | 83 | T=0.3133 | T=0.2286 | 1 | 1.53(1.14-2.07) | **0.004** |
|  |  |  |  | C=0.6867 | C=0.7714 |  |  |  |
| **5** | rs11615 | ERCC1 | 81 | A=0.4768 | A=0.4506 | 0.91 | 1.11(0.84-1.45) | 0.44 |
|  |  |  |  | G=0.5232 | G=0.5494 |  |  |  |
|  |  |  |  | G=0.6329 | G=0.7081 |  |  |  |

**Bibliography**

1 Ward, L. D. & Kellis, M. HaploReg: a resource for exploring chromatin states, conservation, and regulatory motif alterations within sets of genetically linked variants. *Nucleic acids research* **40**, D930-934, doi:10.1093/nar/gkr917 (2012).

2 Rosenbloom KR, S. C., Malladi VS, Dreszer TR, Learned K, Kirkup VM, Wong MC, Maddren M, Fang R, Heitner SG, Lee BT, Barber GP, Harte RA, Diekhans M, Long JC, Wilder SP, Zweig AS, Karolchik D, Kuhn RM, Haussler D, Kent WJ. . ENCODE annotation data:. *Nucleic Acids Res. 2013 Jan;41(Database issue):D56-63. ENCODE data in the UCSC Genome Browser: year 5 update.* .

3 Satterlee, J. S. *et al.* The NIH Common Fund/Roadmap Epigenomics Program: Successes of a comprehensive consortium. *Science advances* **5**, eaaw6507, doi:10.1126/sciadv.aaw6507 (2019).

4 Heramb, C. *et al.* Ten modifiers of BRCA1 penetrance validated in a Norwegian series. *Hereditary cancer in clinical practice* **13**, 14, doi:10.1186/s13053-015-0035-0 (2015).

5 Zhang, J. *et al.* CYP19A1 gene polymorphisms and risk of lung cancer. *The Journal of international medical research* **41**, 735-742, doi:10.1177/0300060513477291 (2013).

6 Oliveira, C. *et al.* Polymorphisms of glutathione S-transferase Mu 1 (GSTM1), Theta 1 (GSTT1), and Pi 1 (GSTP1) genes and epithelial ovarian cancer risk. *Disease markers* **33**, 155-159, doi:10.3233/dma-2012-0920 (2012).

7 Zhou, H. Y., Pon, Y. L. & Wong, A. S. Synergistic effects of epidermal growth factor and hepatocyte growth factor on human ovarian cancer cell invasion and migration: role of extracellular signal-regulated kinase 1/2 and p38 mitogen-activated protein kinase. *Endocrinology* **148**, 5195-5208, doi:10.1210/en.2007-0361 (2007).

8 Gao, N., Nester, R. A. & Sarkar, M. A. 4-Hydroxy estradiol but not 2-hydroxy estradiol induces expression of hypoxia-inducible factor 1alpha and vascular endothelial growth factor A through phosphatidylinositol 3-kinase/Akt/FRAP pathway in OVCAR-3 and A2780-CP70 human ovarian carcinoma cells. *Toxicology and applied pharmacology* **196**, 124-135, doi:10.1016/j.taap.2003.12.002 (2004).

9 Antico-Arciuch, V. G., Dima, M., Liao, X. H., Refetoff, S. & Di Cristofano, A. Cross-talk between PI3K and estrogen in the mouse thyroid predisposes to the development of follicular carcinomas with a higher incidence in females. *Oncogene* **29**, 5678, doi:10.1038/onc.2010.308 (2010).

10 Campbell, I. G. *et al.* Mutation of the PIK3CA gene in ovarian and breast cancer. *Cancer research* **64**, 7678-7681, doi:10.1158/0008-5472.can-04-2933 (2004).

11 Goetz, R. & Mohammadi, M. Exploring mechanisms of FGF signalling through the lens of structural biology. *Nature reviews. Molecular cell biology* **14**, 166-180, doi:10.1038/nrm3528 (2013).

12 Smith, G. *et al.* Individuality in FGF1 expression significantly influences platinum resistance and progression-free survival in ovarian cancer. *British journal of cancer* **107**, 1327-1336, doi:10.1038/bjc.2012.410 (2012).

13 Cole, C. *et al.* Inhibition of FGFR2 and FGFR1 increases cisplatin sensitivity in ovarian cancer. *Cancer biology & therapy* **10**, 495-504 (2010).

14 Sawers, L. *et al.* Glutathione S-transferase P1 (GSTP1) directly influences platinum drug chemosensitivity in ovarian tumour cell lines. *British journal of cancer* **111**, 1150-1158, doi:10.1038/bjc.2014.386 (2014).

15 Henderson, C. J. *et al.* Increased skin tumorigenesis in mice lacking pi class glutathione S-transferases. *Proceedings of the National Academy of Sciences of the United States of America* **95**, 5275-5280 (1998).

16 Yoshihama, T. *et al.* GSTP1 rs1695 is associated with both hematological toxicity and prognosis of ovarian cancer treated with paclitaxel plus carboplatin combination chemotherapy: a comprehensive analysis using targeted resequencing of 100 pharmacogenes. *Oncotarget* **9**, 29789-29800, doi:10.18632/oncotarget.25712 (2018).

17 Qiu, J. *et al.* Association between polymorphisms in estrogen metabolism genes and breast cancer development in Chinese women: A prospective case-control study. *Medicine* **97**, e13337, doi:10.1097/md.0000000000013337 (2018).

18 Pei, X. H., Yang, Z., Lv, X. Q. & Li, H. X. Genetic variation in ERCC1 and XPF genes and breast cancer risk. *Genetics and molecular research : GMR* **13**, 2259-2267, doi:10.4238/2014.March.31.6 (2014).

19 Selvakumaran, M., Pisarcik, D. A., Bao, R., Yeung, A. T. & Hamilton, T. C. Enhanced cisplatin cytotoxicity by disturbing the nucleotide excision repair pathway in ovarian cancer cell lines. *Cancer research* **63**, 1311-1316 (2003).

20 Walsh, C. *et al.* *ERCC5 Is a Novel Biomarker of Ovarian Cancer Prognosis*. Vol. 26 (2008).

21 Walsh, C. S. *et al.* ERCC5 is a novel biomarker of ovarian cancer prognosis. *Journal of clinical oncology : official journal of the American Society of Clinical Oncology* **26**, 2952-2958, doi:10.1200/jco.2007.13.5806 (2008).

22 Khrunin, A. *et al.* Pharmacogenomics of cisplatin-based chemotherapy in ovarian cancer patients of different ethnic origins. *Pharmacogenomics* **13**, 171-178, doi:10.2217/pgs.11.140 (2012).

23 He, J. *et al.* Genetic variants in the nucleotide excision repair pathway genes and gastric cancer susceptibility in a southern Chinese population. *Cancer management and research* **10**, 765-774, doi:10.2147/cmar.s160080 (2018).

24 Chen, J. *et al.* Association of ERCC1 Polymorphisms with the Risk of Colorectal Cancer: A Meta-Analysis. *Critical reviews in eukaryotic gene expression* **27**, 267-275, doi:10.1615/CritRevEukaryotGeneExpr.2017019713 (2017).

25 Pongsavee, M. & Wisuwan, K. ERCC5 rs751402 polymorphism is the risk factor for sporadic breast cancer in Thailand. *International journal of molecular epidemiology and genetics* **9**, 27-33 (2018).

26 Li, X., Zhang, R., Liu, Z., Li, S. & Xu, H. The genetic variants in the PTEN/PI3K/AKT pathway predict susceptibility and CE(A)F chemotherapy response to breast cancer and clinical outcomes. *Oncotarget* **8**, 20252-20265, doi:10.18632/oncotarget.15690 (2017).

27 Hua, K. *et al.* Estrogen and progestin regulate metastasis through the PI3K/AKT pathway in human ovarian cancer. *International journal of oncology* **33**, 959-967 (2008).

28 Yang, X. *et al.* Association of the functional BCL-2 rs2279115 genetic variant and small cell lung cancer. *Tumour biology : the journal of the International Society for Oncodevelopmental Biology and Medicine* **37**, 1693-1698, doi:10.1007/s13277-015-3934-9 (2016).

29 Yang, X. *et al.* Association of the functional BCL-2 rs2279115 genetic variant and small cell lung cancer. *Tumor Biology* **37**, 1693-1698, doi:10.1007/s13277-015-3934-9 (2016).

30 Pan, W. *et al.* Functional BCL-2 regulatory genetic variants contribute to susceptibility of esophageal squamous cell carcinoma. *Scientific reports* **5**, 11833, doi:10.1038/srep11833 (2015).
